# Francisco José de Goya y Lucientes (1746–1828). Cat Fight (1786–1788).

**DOI:** 10.3201/eid0911.AC0911

**Published:** 2003-11

**Authors:** Polyxeni Potter

**Affiliations:** * Centers for Disease Control and Prevention, Atlanta, Georgia, USA

**Figure:**
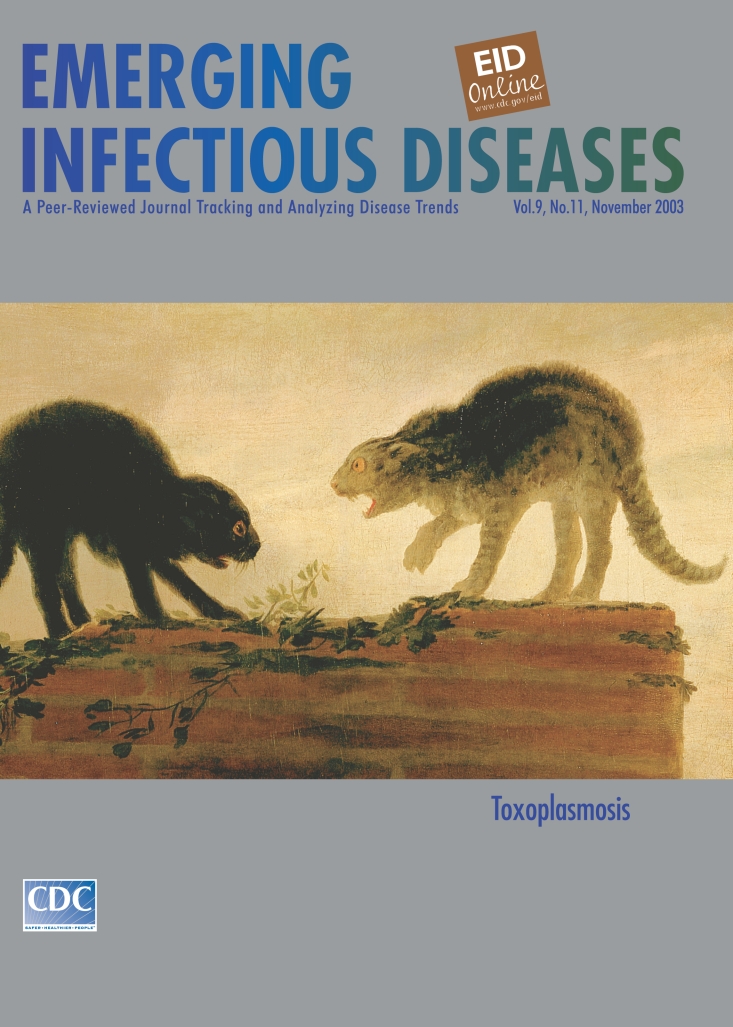
Francisco José de Goya y Lucientes (1746–1828). Cat Fight (1786–1788). Model for tapestry intended to decorate the dining room of the Prince of Asturias. 1.40 m x 4.38 m Museo Nacional Del Prado, Madrid, Spain

**Table:** 

'Imagination abandoned by reason produces impossible monsters; united with her, she is the mother of the arts,"wrote Goya in the subtitle of his etching The Sleep of Reason Produces Monsters. Imagination, its origins and its limits, fascinated Goya and permeated his work, outweighing tradition and ranking him 'first of the moderns."Fantasy and invention marked the course of a career during which he revolutionized tapestry design, excelled in portraiture, and along with Dürer, Rembrandt, and Whistler, became one of the greatest graphic artists of all time (*1*).

Goya was born in the village of Fuendetodos near Zaragoza, in northern Spain, the son of an altar gilder. At 14, he was apprenticed to a local painter and then traveled to Italy, where he learned the decorative manner of rococo. Early in his career, as designer for the royal factory in Madrid, he turned his keen observations of human behavior into innovative tapestry designs depicting scenes of everyday life. He also favored bold new techniques in oil, fresco, and drawing. Influenced by neoclassicism and the works of Velázquez, he moved away from the Baroque toward a realistic portrayal of his time, which he enriched with incisive satire, fantastic visions, and his own interpretation of human nature (*2*).

Goya was an enigmatic figure. The painter of three generations of kings, he mastered charm and diplomacy, was linked to power, and achieved popular success. Yet, he remained an outsider, often at odds with the court, the monarchy, and the church. His royal portraits were filled with candor and honesty to the point of caricature, his religious subjects were ambiguous and marked by earthy realism, and his women in the flesh caught the attention of the Spanish Inquisition. Many of his original prints, which influenced such masters as Delacroix, Manet, and Picasso, were not seen until after his death because they were deemed too critical of the political and religious order of his day.

Sweeping life changes over Goya's 60-year artistic career are reflected in his work, whose pioneering emotional content influenced the course of 19th and 20th century art. The Spain in which he had been successful without rival in his early years disappeared during the Napoleonic wars. Seven of his children died before reaching adulthood. And serious illness, compounded by his wife's death and other adversities, left him weak, disillusioned, and destitute near the end of his life (*3*).

Goya's illness, perhaps saturnism caused by toxic fumes from lead salts in the paint he used, brought chronic headaches and permanent hearing loss. Overcome with pessimism, isolated, and traumatized, he created his own aesthetic, turning personal demons into deadly universal themes and painting horrific fantasies laced with caustic social commentary. A collection of 84 prints (Caprices, 1799) satirized the clergy, the nobility, and society's foibles and vices, not the least of them superstition, ignorance, and fear. Another series of 82 realistic etchings (Disasters of War, 1810–1814) chronicled the atrocities following the violence and devastation of the Napoleonic invasion of Spain (*1*).

Goya was an artist of contradictions and opposites. Along with the bizarre, he painted the comical, along with darkness, bright light. As a result, his images provoke at once fear and delight, sadness and ghoulish mirth. With growing pessimism, he painted bizarre scenes populated with outlandish creatures and monsters engaged in witchcraft, cannibalism, and carnage. Demons, lunatics, lynxes, corpses, and gore allude to human preoccupation with death (*4*). Goya's imagination, without abandoning reason, harnessed the supernatural into a troubling display of the unconscious and the irrational.

Animals, embodying human weaknesses, social tension, tragedy, the premonition of death, or some other insidious danger, appear repeatedly on a stage of constant hostility and conflict. At their most menacing, they exercise extraordinary powers and influence. In the Cat Fight, on this month's cover of Emerging Infectious Diseases, bellicose cats, frozen in time, apropos of nothing, engage the spectator through pity and terror.

Perched it seems on the edge of the world against an eerie void, these wild creatures stand in electrifying anticipation of trouble. Puffed, curled, glowering, almost translucent against the airless, depthless, oppressive space, they have lost their natural self-directedness. Like marionettes in the hands of an expert puppeteer, they seem to float in fluorescent space, awaiting instructions for the next move in what is certain to be a bloody contest. The brilliant yellow and red highlights reinforce the dreamlike unreality of the scene (*4*).

Goya's exquisite pessimism, brought on by debilitating illness, guided his exploration of the fundamental mystery of human behavior. Contemporary investigations of the biologic as well as psychological motivators of behavior are guided by research, which now implicates infectious agents in what was long thought 'madness."For example, *Toxoplasma gondii*, a single-celled parasite that begins and completes its life cycle in domestic cats, can alter behavior in animals and produce psychotic symptoms in humans (*5*). *T. gondii*, which causes toxoplasmosis in cats and other mammalian species, may be contributing to some cases of schizophrenia.
